# Calling All Curators: A Novel Approach to Individualized Interactive Instruction

**DOI:** 10.5811/westjem.2017.11.35239

**Published:** 2017-12-14

**Authors:** Gita Pensa, Jessica Smith, Kristina McAteer

**Affiliations:** Alpert Medical School of Brown University, Department of Emergency Medicine, Providence, Rhode Island

## Abstract

With the increasing influence of the “Free Open Access Medical Education” (FOAM or FOAMed) movement, it is critical that medical educators be engaged with FOAM in order to better inform and direct their learners, who likely regularly consume these materials. In 2012, the Accreditation Council for Graduate Medical Education (ACGME)/Residency Review Committee (RRC) began to permit 20% of emergency medicine (EM) residents’ didactics hours to be earned outside of weekly conference, as “Individualized Interactive Instruction” (III) credits.^1^ We describe a digital course in EM, “Asynchrony,” as an approach to FOAM to meet these III standards. Asynchrony is geared toward EM residents using FOAM and other online learning tools, curated by faculty into narrative, topic-specific educational modules. Each module requires residents to complete a topic assignment, participate in a discussion board, and pass a quiz to earn ACGME-approved III didactic credit; all of this is tracked and filed in an online learning management system.

## INTRODUCTION

The Free Open Access Medical (FOAM) education movement has become an increasing presence in emergency medicine (EM).^2^ Many residents and medical students regularly consume online educational blogs, podcasts, or other digital educational resources, usually without any faculty guidance or oversight.^3^ While some of these resources are peer-reviewed and of very high academic quality, tools for the critical evaluation of these resources are evolving. Many medical educators are concerned that their learners are being influenced by unvetted sources and celebrity purveyors of medical “edutainment.”^4^

Educators who are regularly engaged with FOAM resources may be in a unique position to serve as curators and translators of this world for their trainees. In a recent commentary in the *Canadian Journal of Emergency Medicine*, the authors proposed three types of scholars for this new era of evidence-based medicine: the “critical clinician,” the “translational teachers,” and the “interactive investigators.”^4^ The “translational teachers” include, among others, educators who strive to improve clinical practice of their learners by shortening the “knowledge translation window” – that is, assisting the scientific community in disseminating new knowledge to learners, with the help of modern educational tools such as social media, blogging, or podcasting.^4^

To be a “translational teacher,” one need not create these educational tools from scratch. Curation and presentation of high-quality digital materials to learners under faculty direction can help satisfy millennial learners’ desire for digital, asynchronous and on-the-go learning, while keeping educators from having to re-invent the wheel.

## METHODS

The details of how residency programs could adopt novel Individualized Interactive Instruction (III) opportunities were not clearly delineated by the Accreditation Council for Graduate Medical Education (ACGME). Many residency programs took different approaches. In 2014, our program sought to create a digital course, “Asynchrony,” as a new approach to FOAM and other popular digital content, vetting and interpreting it for our learners, while simultaneously meeting the ACGME’s four criteria for III:^1^

1. The program director must monitor resident participation.
2. There must be an evaluation component.
3. There must be faculty oversight.
4. The activity must be monitored for effectiveness.

### The Asynchrony Course

This is the third year of the course entitled “Asynchrony” at our institution. Using our medical school’s online learning management system (LMS) we designed a faculty-led digital III course in EM, mapped to our residency’s curricular calendar. This course provides faculty perspective for trainees who consume FOAM resources, while allowing residents to earn ACGME-compliant III credit.

Weekly or bi-weekly modules each cover a particular EM educational topic. Each module is divided into an assignment page, a discussion page, and a 10-question, multiple-choice quiz. The assignment page is designed to be completed in one hour (or two hours if it is a longer, bi-weekly module).

### The Assignment Page

Faculty search for, evaluate, and accept or reject applicable clinical content resources in a variety of digital formats (podcasts, blog posts, video posts, radiologic images, journal article links, online textbook entries). Because this course is designed for all post-graduate learners in EM, faculty also aim to evaluate content for applicability to varied levels of learner experience (balancing basic content and more advanced articles/practice controversy).

The curated material is then assembled into an informal, engaging narrative (including a theme song using a play on words related to the content, just for fun). The educational content is accessed through hyperlinks woven into the narrative, and the faculty give commentary on what is assigned when they feel it is necessary.

Because the FOAM world is often lacking in quality material for less “exciting” core content,^5^ faculty may use online journal articles or reviews, podcasted lectures, or links to our university’s e-library to fill in perceived gaps. “Optional Extras” are also included, which can include lay press articles, tangentially related fun facts, links to additional resources, or human-interest stories related to conditions being discussed in the modules.

Some open-access example assignment pages can be viewed on our educational blog at www.brownemblog.com/asynchrony.

### The Discussion Page

Once the assigned content is reviewed and completed by the residents, they navigate to the discussion board. Participation on the discussion board is mandatory, which opens the gate to the quiz. To encourage participation from all levels of learners, several leading queries are provided for the resident to choose; some are general, and others require more advanced expertise. The faculty member monitors and facilitates the discussion; approximately 20 faculty participated on the discussion boards in the first year of the course. Multiple faculty experts may be tapped for answers to resident questions posted on the board.

### The Quiz

The online quiz includes 10 multiple-choice questions, written by the faculty curator for that module. All residents have three tries to pass the same quiz; 8/10 earns them III credit. The credits are logged automatically by the LMS. The LMS also tracks residents’ performance on individual questions, as well as pooled data for all respondents to each question, for troubleshooting purposes.

## RESULTS

Survey data from the first year of use with 33 of our 48 residents responding an overall 4 or 5 on a 1–5 scale (5 being excellent), described the program as follows: on ease of use (mean 4.15); quality of content (mean 4.58); variety of content (mean 4.58); resources used (4.36); and appropriateness of time spent doing the activity (mean 4.03). Some residents felt the modules took too long to complete (per one resident’s survey comment, “very well put together, but they take longer than the one hour of conference they are replacing.”). However, some residents stated they preferred the online format to traditional weekly conference (e.g., “Finally, a place that utilizes the incredible online resources available that are much better done than most presentations.”).

The Asynchrony program is optional, but interestingly, in the survey comments several of the residents requested that it become mandatory (e.g. “They are amazing. I wish we had a structured requirement for its use.”). We currently have five hours of live weekly conference, with Asynchrony as an additional option to use for up to 20% of the annual conference hours requirement.

As one might expect due to the optional nature of the course, there are some residents who engage every week, others who “binge” on several modules every month or two, and others who do not ever participate. Likewise, there are faculty who are active on the discussion boards or interested in creating modules, and others who eschew the electronic format completely.

When queried about barriers to participation, lack of time was the most common reason cited.

Based on survey results, changes this year have included a trial of longer, bi-weekly modules for two hours of III credit, as well as placement of selected assignment pages as open-access material on our blog, which other EM programs may use if desired.

A video example of an actual module within our LMS is available at https://www.youtube.com/watch?v=GLh082URR0k&t=2s.

## CONCLUSION

The Council of Emergency Medicine Residency Directors (CORD) III Task Force released a post on “Best Practices in III” in June 2016.^6^ The advantages of implementing a course similar to Asynchrony include the following:

1. Faculty curation of FOAM/digital resources ensures content quality, and helps faculty stay up-to-date as well.
2. Faculty can fill in gaps not covered by FOAM.^5^
3. Interactive nature fulfills III Best Practices,^6^ gives perspective, and allows residents to ask questions of trusted sources.
4. Residents earn III credit for work they may have already been doing.^3^
5. Material can be assembled into a cohesive, ordered curriculum, unlike the piecemeal manner in which FOAM is normally consumed.
6. Quiz scores, interactions, and credits are tracked via the LMS.

Potential barriers and limitations include these:

1. Commitment – Asynchrony requires a faculty champion (committing several hours per week), time of other faculty contributors, and familiarity with an online LMS
2. Participation – If not mandatory, not all residents or faculty will regularly participate
3. Time constraints – Content required to fully explore a topic may take residents longer than one hour to complete.

Asynchrony aspires to be an informative, entertaining resource for our residents. It would be easily replicable at other institutions and creates a cohesive system integrating multi-media digital learning and FOAM into residency education.
